# The Health of School Children

**Published:** 1911-11-11

**Authors:** 


### THE HEALTH OF SCHOOL CHILDREN.

### Sir George Newman's Report.

(Reviewed by a Medical Inspector.)

The recently issued report of the Chief Medical Officer of the Board of Education "is an account and, to some extent, a criticism of many different kinds of work—work done in areas so various and in ways so different that comparison is difficult and general statement precarious, if not illegitimate." This is a wise view. In many fields, in or outside medicine, a great amount of labour has been spent and is—it is to be feared—still spent on drawing up statistics and in arriving at conclusions from statistics which have very little value, or may have less than no value, because they form a basis for erroneous action. The report in question purports, therefore, to throw out suggestions rather than lay down laws. Amongst many other subjects in this voluminous report the vexed question of the diagnosis of pulmonary tuberculosis in children is dealt with.

### Phthisis in School Children.

Sir G. Newman remarks: "From recent discussions, and from papers dealing with this subject, it would appear that the opinion of a considerable section of medical practitioners is slowly undergoing a change. It is fully admitted that the typical, well-defined signs of phthisis, as they exist in the adult, are not frequently found in school children; but many physicians now consider that pulmonary tuberculosis runs a somewhat different course in childhood and in adult life." Later it is added "in children it is probable that the affection of the lung is usually secondary to disease of the mediastinal or bronchial glands." With these statements little fault can be found, yet it is no new discovery to physicians or pathologists connected with children's hospitals that "typical well-defined signs of phthisis" are rare in childhood. A great number of school medical inspectors, possessing little or no knowledge of children's diseases, commenced work with the belief that they would find what they had been accustomed to find in adult patients, and it is one of the unfortunate defects of human investigation that it too often finds what it expects to find.

### Diagnostic Signs of Tuberculosis in Children.

The gentle reference by Sir George Newman to disease of the bronchial glands is no doubt diplomatic. Any medical man possessed of a wide knowledge concerning matters affecting public health must know that for many years the main source of tubercular infection in children has been stated by morbid anatomists to be through the bronchial glands. This well-known fact is no doubt judiciously brought to the notice of the many inspectors who will read the report as a comparatively recently accepted opinion. The employment of Von Piquet's test in the diagnosis of tuberculosis is referred to, and no great stress laid upon its reliability. Such an attitude is no doubt correct. Whatever may be the value of the test as an indication that at some time in the child's history tuberculous infection has occurred, its value must be slight in determining whether a child is or is not suffering from active tuberculosis. While, however, the report tends to maintain the view that discoverable tuberculosis of chronic nature within the lungs is rare, the reasonable attitude is adopted that poorly-nourished, delicate children should be dealt with as if likely to develop phthisis, and many useful suggestions are made with regard to observation and treatment of such children.

In connection with diagnosis of various affections, it may perhaps here not inappropriately be indicated that care is needed in the diagnosis of heart disease, not so much in omitting to notice its presence when it really exists as in expressing an opinion that it exists when it does not. An error of this kind may cause unnecessary disturbance of the peace of mind of parents and child, and not only affect a child's outlook on life but influence his character throughout his future career. Of less importance connected with diagnosis, yet a matter of distinct interest and importance, is the testing of children's blood for the percentage of hæmoglobin in the cases admitted to open-air schools, upon which the report lays stress. It is to be feared that the method usually employed in these schools is very unsatisfactory and leaves much scope for the imagination, however conscientious the examiner may endeavour to be. Reports of great improvement in the condition of the blood during the months anæmic children attend open-air schools it is to be hoped are true, but in this matter, as in many others, there is considerable risk of mistaken observation.

### Open-air Education.

This remark is not intended to cast any slur upon open-air schools. It is necessary, however, to guard against basing apparent results obtained on insufficient grounds. Not only open-air schools, but work conducted as much as possible in the open air, is recommended, and any move towards this end cannot but be beneficial in strengthening powers of resistance to disease. The report is interesting as showing how much is being done in the direction not only of the establishing of various means for giving instruction in the open air, but in conducting school camps for London children in various places. It is mentioned "that the object of the camps and school journeys is recreative and educational rather than ameliorative, but the value of the open-air life and the lessons in practical hygiene to be learnt on such journeys make the camps a valuable adjunct to open-air education." This is no doubt true. A love for the open country instilled into the minds of school children must have some effect in producing dissatisfaction with the overcrowded condition of many of our cities. The children of to-day are the voters of to-morrow, and in their hands to a great extent will be the future of social reform.

Epileptic Children.

On schools for various forms of defective children the report has a few brief words. Amongst the most needy are probably the schools for epileptic children. Expensive though no doubt such residential schools are, the establishing of a larger number would fill a great want. Potassium bromide is spoken of as the drug employed in these institutions. This drug is, needless to say, the drug of all others in the treatment of epilepsy, yet it may be worth while mentioning that occasionally, at least for a time in children, borax is a useful remedy. Kindred institutions—those for the feeble-minded—are also dealt with and stress laid upon the "after-care" of children admitted to them. It scarcely needs to be pointed out that it is important to ascertain how far the education of the feeble-minded is justified by results. Results will obviously depend largely upon the standard of feeble-mindedness adopted by those who are responsible for admission to these schools. Personal judgment, here as elsewhere, influences the value of statistics. However this may be, it may be assumed that all children transferred to schools for the mentally defective have been found to be very difficult to educate in ordinary schools. This fact being admitted, it is interesting to note that of 211 girls leaving eight schools for the mentally defective under the London County Council as many as 51.2 per cent. were "in good and promising work" from seven to seventeen years after leaving school. In Birmingham the percentage was considerably lower, and also in Liverpool; but even there, judged by practical results alone, they are probably worth the effort and expense. Allowing, however, that the results are not commensurate with the expense, children who cannot be educated in an ordinary school need some kind of supervision and care. Many probably will meet with little wise discipline in their own homes, whereas in the schools they will at least learn something towards realising that they are members of a community, and that its other members are doing something to improve their condition.

Treatment.

As might be expected, treatment of various remedial defects is somewhat fully dealt with, and stress is laid upon the necessity of education committees considering this subject. The report goes so far as to say that "a school medical service which stops short of organised treatment, direct or indirect, is incomplete and does not yield a fair return on the expenditure incurred." Attention is also drawn to the frequency of malnutrition and to the great "physical handicap" it is to school children. Anyone familiar with school children must agree with such a statement. These pale, lethargic, often more or less wasted children, present also puzzling questions from the point of view of etiology. As Sir George Newman states, "merely to increase the supply of food would in many cases not solve the complex problem." One member of a family may be of this type, when all the rest are ruddy and robust. Yet no doubt, as the report states, deficient food is frequently an important, if not an all-important, factor. In conclusion it may be remarked that the report is instructive, suggestive, and takes a broad view of the various questions which must exercise the minds of those responsible for the administration of medical inspection of schools.

### SUMMARY OF THE REPORT.

The report, which is full of most instructive details, shows that defective nutrition stands in the forefront as the most important of all physical defects from which school children suffer. In London, for instance, not less than 11 per cent. of the children examined were found to be suffering from a greater or lesser degree of malnutrition. Uncleanliness as evidenced by the presence of head vermin is indicated generally by saying that in ten rural counties taken at random 70 per cent. of the girls' heads were clean, whereas in ten typically urban communities 51 per cent. of the girls' heads were found to be clean. It is satisfactory to note, however, that since the introduction of medical inspection the evil of uncleanliness has undoubtedly been lessened. The important question of defective vision is receiving increasing attention, and there is evidence from the reports from different parts of the country that a real effort is being made in many areas to remedy the serious defects which medical inspection has revealed. The frequency with which adenoid growths are found varies greatly in different areas, partly because the conditions which lead to their development are more commonly met with in certain areas, and partly because some medical officers are more careful in their observation and estimation of defects than others; in the urban areas of Aston Manor, Darlington, and Luton the percentage with adenoids is returned as approximately 16, while in the urban area of Shrewsbury the percentage is .85. So far as the curtailment of ringworm is concerned, but little appreciable headway seems to have been made during the year. Dealing with the treatment of ringworm by the *x*-ray method, the report says that the best results are reported from Bradford, where the treatment has been carried out on a large scale. The average length of time between exposure and return to school has been thirty-three days. The report deals at some length with the existing provision for the treatment of tuberculous children, and suggestions for treatment by the local Education Authority. The report further deals with medical treatment, with school clinics, and with defective and epileptic children, education of feeble-minded children, open-air education, and other kindred topics. It is not possible to summarise a report of this nature, but roughly it is estimated that out of six million children registered on the books of the public elementary schools of England and Wales about 10 per cent. suffer from a serious defect in vision, from 3 to 5 per cent. suffer from defective hearing, 1 to 3 per cent. have suppurating ears, 6 to 8 per cent. have adenoids or enlarged tonsils of sufficient degree to obstruct the nose or throat, and thus to require treatment; about 40 per cent. suffer from extensive and injurious decay of the teeth, about 30 to 40 per cent. unclean heads or bodies, about 1 per cent. suffer from ringworm, 1 per cent. from tuberculosis in readily recognisable form, from 1 to 2 per cent. are afflicted with heart disease, and a considerable percentage of children are suffering from a greater or less degree of malnutrition.

